# Aging remodels progenitor and niche interactions in lung regeneration

**DOI:** 10.3389/fragi.2026.1986986

**Published:** 2026-10-28

**Authors:** Fernanda Toscano-Marquez, Angeles Garcia-Vicente, Uriel Camacho-Silverio, Tania Valdivia-Herrera, Mariana Río de la Loza, Elisa Hernández-Xóchihua, Remedios Ramírez, Moises Selman, Annie Pardo, Yair Romero

**Affiliations:** 1 Instituto Nacional de Enferemedades Respiratorias “Ismael Cosio Villegas”, Mexico City, Mexico; 2 Facultad de Ciencias, Universidad Nacional Autónoma de Mexico, Mexico City, Mexico

**Keywords:** autophagy, lung organoids, mTOR, nuclear envelope, senescence, ZMPSTE24

## Abstract

**Introduction:**

Progenitor cells in aged tissues undergo changes in their microenvironment that may impact their functionality during regeneration. Despite recent advances in understanding the role of adult lung progenitors, the impact of aging on these cells remains unclear.

**Methods:**

To analyze aging modifications, we used aged wild-type mice of 18–24 months old, and Zmpste24^–/–^ deficient mice, which exhibit an accelerated phenotype. A three-dimensional organoid culture system was employed to assess *in vitro* regenerative capacity. Additionally, cells were isolated and characterized with senescence and autophagy markers.

**Results:**

Our findings revealed that lung epithelial cells from aged mice and Zmpste24^–/–^ mice retain organoid forming potential, maintaining their phenotype and cellular homeostasis through increased autophagy-associated transcriptional response, particularly when co-cultured with young fibroblasts. Moreover, these progenitor cells may exhibit increased susceptibility to mechanical stress with aging potentially associated with reduced nuclear lamins and the Zmpste24 defect. This vulnerability is illustrated by FACS sorting, which can further compromise their regenerative potential. By contrast, cultured fibroblasts from Zmpste24^–/–^ mice show nuclear defects and acquire a senescent phenotype, characterized by the mechanistic target of rapamycin complex 1 (mTORC1) activation and reduced autophagy, which in turn impairs organoid formation.

**Discussion:**

Our results indicate that, in aging, progenitor cells and their fibroblast niche integrate microenvironmental signals that shape cell-cell interactions essential for lung regeneration.

## Introduction

The aging phenotype is associated with a limited regenerative capacity, mainly due to stem cell exhaustion. The lung is considered a low-turnover organ, where regeneration depends on the activity of rare stem cells and facultative progenitors (Alysandratos et al., 2021). In the airway epithelium, basal cells have been identified as rare stem cells responsible for its regeneration (Rock et al., 2009). In the alveolar region, regeneration is primarily mediated by facultative progenitors, particularly a subpopulation of alveolar epithelial type 2 (AT2) that can restore alveolar structure through the interaction with a single-cell niche composed of fibroblasts (Nabhan et al., 2018; Zacharias et al., 2018; Barkauskas et al., 2013). Crucially, in the alveolar epithelium such cell–cell interactions influence cell fate decisions (Liberti and Morrisey, 2021). Considering the central importance of such interactions in orchestrating regenerative processes, it is therefore essential to determine whether alterations associated with aging prevent lung regeneration and promote stem cell exhaustion.

Despite recent advances in understanding adult lung stem cells, the effect of aging on their function remains largely unknown. To address this gap, we compare physiological and accelerated aging using distinct mouse models: Zmpste24-deficient mice (a model of premature aging), physiologically aged wild-type mice, and young wild-type mice controls. Zmpste24 is a metalloprotease critical for lamin A/C processing. Although Zmpste24-deficient mice do not exhibit an aging phenotype at early stages, by 16 weeks they display hallmark features of premature aging, such as weight loss, graying hair, and reduced lifespan compared to wild-type counterparts (Varela et al., 2005; Pendás et al., 2002). This model thus provides a valuable tool to explore whether accelerated aging recapitulates key aspects of physiological lung aging.

Lopez-Otín, *et al.* have proposed that among the hallmarks of aging, stem cell exhaustion and altered intercellular communication are key determinants of the aging phenotype. Although all hallmarks are interconnected, they can be conceptually organized as a sequence of events. Initially, primary features emerge, such as DNA damage, epigenetic changes, loss of proteostasis and impaired autophagy (López-Otín et al., 2013; López-Otín et al., 2023). Among these, defective autophagy is particularly critical, as it comprises stem cells function and survival (Hansen et al., 2018). Autophagy maintains cellular homeostasis by removing damage or unnecessary cytoplasmic components through the lysosomal pathway (Kroemer, 2015).

In later stages, certain responses act as defense mechanisms but may become detrimental when dysregulated. These include antagonistic processes such as cellular senescence and autophagy since in the context of aging, autophagy prevents cellular senescence by preserving stem cell function and viability promoting regeneration (Liu and Sabatini, 2020; García-Prat et al., 2016; Yilmaz et al., 2012). The mTOR pathway serves as a central regulator, integrating signals to modulate stem cell potential in a tissue-specific manner (Sousa-Victor et al., 2014).

Therefore, the aim of this study was to determine whether age-associated alterations in nuclear envelope integrity, autophagy, cellular senescence, and the fibroblast niche contribute to lung progenitor cell exhaustion and the decline in regenerative capacity observed during aging.

## Materials and methods

### Murine model

Animal use complied with relevant guidelines and regulations for animal care, and the protocol was approved by the Bioethics Committee of the Instituto Nacional de Enfermedades Respiratorias “Ismael Cosio Villegas” (Approval Codes: B01–2023 and B17-25). C57BL/6J mice (wild type) of 12–16 weeks and 18–24 months were used as young and physiologically aged groups, respectively. Zmpste24^−/−^ mice were used as a model of accelerated aging; animals were included from 16 weeks of age, and both genotyping and phenotype characteristics were confirmed prior to inclusion (Supplementary Figure S1). This strain was developed and kindly donated by Dr. Carlos López-Otín.

### Isolation of lung epithelial cells and fibroblasts

Lungs of WT and Zmpste24^−/−^ mice were perfused with Phosphate-Buffered Saline 1X (PBS) and instilled with dispase (Corning # 47743) for 45 min at room temperature. Trachea and upper airways were removed. The lung was mechanically dissociated and filtered through 70μm and 40 μm nylon filters. After Red Blood Cell Lysis Buffer was used. The cell suspension was incubated for epithelial cell positive selection with anti-mouse CD326 (EpCAM+) attached with magnetic beads (Miltenyi Biotec #: 130-105-958). Epithelial cells were used immediately, and purity was determined by RT-qPCR of surfactant protein C (SPC). Lung fibroblasts cells were obtained from the negative fraction. Cells were plated on, and media was changed 24h after to remove cells that were not attached (non-mesenchymal). Fibroblasts were maintained in culture with DMEM medium with 5% FBS in a 37 °C incubator in an atmosphere of 95% air and 5% CO_2_ until use.

For some experiments cells were starved in Hank’s Balanced Salt Solution (HBSS) (Gibco, #14025092) for 3 h, then stimulated with complete medium for 24 h in the presence of 10 μM chloroquine (Sigma, #C6628) to induce mTORC1 activation and block lysosomal degradation, finally fibroblasts were washed with fresh culture medium.

#### Lung organoid culture

For lung organoid culture, freshly harvested epithelial cells (EpCAM+) were cocultured with fibroblasts; in a ratio of 1,000:10,000 (epithelial: fibroblast). Fibroblasts and epithelial cells were isolated from different mice, with no pooling between mice, such that 1 mouse represents one biological replicate. Experiments were performed in independent isolation rounds with a total of two animals per group, the corresponding n values, indicating the number of biological replicates, are included in each figure. Fibroblasts from young and aged mice were isolated on preceding days and expanded up to passage three prior to being used to ensure adequate culture purity. Epithelial cells were seeded in 2–4 transwells per animal as technical replicates. Briefly, epithelial cells and fibroblasts were co-cultured in a 1:1 Matrigel–culture medium mixture (Corning, Cat. #356237, Lot #3061001). MEMα-based medium (Gibco, Catalog # M4526) was supplemented with 10% Fetal Bovine Serum (FBS), 1× insulin/transferrin/selenium, 2 mM L-glutamine, penicillin-streptomycin, 0.25 μg/mL amphotericin B, 0.0002% heparin and ROCK pathway inhibitor at 1 µM (Selleckchem, Catalog # S1049). The 6.5 mm transwell with 0.4 μm pore polyester membrane inserts (Corning, Catalog # CLS3470) were required for culture to establish an air-liquid interface. Organoids were cultured with media changes every 2 days and fixed using Fixation Buffer (Sigma, Catalog # F1797). Since organoids are randomly distributed across different focal planes within the Matrigel, we first examined the entire z-stack to identify the focal plane containing the highest number of visible organoids. This same focal plane was then used for organoid quantification across all experimental conditions. Thus, the focal plane was selected using a common criterion for the entire experiment rather than independently for each experimental group. Photographs of bright fields were taken using an inverted microscope (Nikon) and images were analyzed with NIS-Elements Software.

### Flow cytometry

After separation with magnetic beads, Ep-CAM-positive cell suspensions were stained with anti-CD326 PE-Cy7 antibody (BioLegend) for 30 min at 4 °C, avoiding light. They were subsequently washed and subjected to fluorescence-activated cell sorting. Using a FACS Aria II (BD), cells were identified by gating singlets, and CD326+. Double-selected (isolated with magnetic beads and FACS) Ep-CAM-positive cells were cultured to obtain organoids.

### Senescence associated-β galactosidase assay

Senescence associated β-galactosidase (SA-β-gal) staining was performed in fibroblasts and transwell-inserts containing organoid cultures with the Senescence Cells Histochemical Staining Kit (Sigma, Catalog # CS0030) according to the manufacturer’s instructions.

### RT-qPCR

RNA of epithelial cells was isolated using Trizol reagent (Invitrogen) and cDNA was synthesized from 500 ng of RNA using iScript cDNA Synthesis Kit (Bio-Rad) according to manufacturer’s protocol. Quantitative PCR amplification was performed in a mixture containing 6 ng of cDNA, and the following primers: CDKN1A Rv:CCTGTTCTAGGCTGTGACTGCTT; Fwd: CAT​TCC​CTG​CCT​GGT​TCC​TT, and p53 Rv: GGCGAA AAGTCTGCCTGTCTT, Fwd: CGC​TGC​TCC​GAT​GGT​GAT, β-actin Rv: GGC​TGT​ATT​CCC​CTC​CAT​CG, β-actin Fwd: CCA​GTT​GGT​AAC​AAT​GCC​ATG​T. In addition, TaqMan probes were used for SPC and 18s quantification. Thermocycler QS6Flex (Applied Biosystems) was used for qPCR experiments, ΔCt was used for data analysis.

Gene expression analysis of mouse autophagy-related genes was performed using the RT^2^ Profiler PCR Array Mouse Autophagy (PAMM-084Z, Qiagen) and processed with the RT^2^ Profiler PCR Data Analysis tool (GeneGlobe, Qiagen). cDNA samples were obtained from epithelial cells of four KO mice and four WT mice. Ct values were normalized to the arithmetic mean of the housekeeping genes (Actb, B2m, Gapdh, and Gusb).

#### Immunocitochemistry

Paraffin-embedded organoid sections (3 µm) were prepared for immunohistochemistry (IHC). Antigen retrieval was performed using a citrate buffer, followed by permeabilization and blocking. Sections were incubated overnight at 4 °C with the primary antibody against proSPC (Millipore, Catalog #AB3786). After washing, samples were incubated with the appropriate secondary antibody, followed by horseradish peroxidase–conjugated streptavidin, and counterstained with hematoxylin. Images were acquired using an inverted microscope (Eclipse, Nikon) and analyzed with NIS-Elements software.

#### Immunofluorescence

Lungs were perfused with PBS 1X to remove blood, fixed with 10% neutral buffered formalin (NBF), and embedded in paraffin blocks. Antigen retrieval was performed using a citrate buffer, followed by permeabilization and blocking. Sections were incubated overnight at 4 °C with the primary antibody against Lamin A/C (GeneTex, Catalog #GTX101127). After primary antibody incubation, Alexa Fluor 546 donkey anti-rabbit secondary antibody (Invitrogen, Catalog #A10040) was applied. Nuclei were counterstained with 4′,6-diamidino-2-phenylindole (DAPI) (Sigma Catalog #D1306). EpCAM+ cells were seeded on coverslips coated with Matrigel and incubated for 2 h. Cells were then fixed with 4% paraformaldehyde for 20 min at room temperature, followed by two washes with 1X PBS. Blocking and permeabilization were performed with 0.5% Triton X-100 in PBS containing 5% BSA for 45 min. Cells were incubated overnight at 4 °C with the following primary antibodies: SPC (Millipore, Catalog #AB3786), HOPX (Santa Cruz Biotechnology, Catalog # sc398703), Anti-Podoplanin/T1alpha Syrian hamster monoclonal antibody (Abcam, # ab11936), Ki67 (Abcam, Catalog # ab833), and H2AX (Abcam, Catalog #ab11174). The corresponding secondary antibodies were Alexa Fluor 488 donkey anti-rabbit (Invitrogen, Catalog #A-21206) or Alexa Fluor 549 donkey anti-mouse (Invitrogen, Catalog #A-21203). Nuclei were counterstained with DAPI (Catalog #D306). Images were obtained using confocal Leica TCS-SP8.

### Western blot

Proteins were extracted from cells using RIPA lysis buffer (Sigma Catalog #R0278), and 20 μg of total protein was used in polyacrylamide gels for electrophoresis. Nitrocellulose membrane was used to transfer proteins and block for 1 h. Primary antibodies for LC3 (Sigma, Catalog #L7543), P62 (Abcam, Catalog #ab56416), S6 (CST, Catalog #2217), p (235/236)S6 (CST, Catalog #2211) Lamin b1 (Invitrogen, Catalog #702972), Lamin A/C (GTX, Catalog #101127) and β-actin (Sigma, Catalog #A5441) or H3 (GTX, Catalog #122150) as a loading control were incubated overnight. Finally, Li-Cor infrared secondary antibodies and Odyssey scanner (Li-Cor) were used. Quantification was done using ImageJ software (NIH).

### Statistical analysis

Data are presented as mean ± standard deviation. When the assumptions of normality and homogeneity of variances were satisfied, statistical comparisons were performed using either a two-tailed Student’s t-test (for comparisons between two groups) or one-way analysis of variance (ANOVA) followed by Tukey’s *post hoc* test (for comparisons among more than two groups). A *p*-value of less than 0.05 was considered statistically significant. All statistical analyses were conducted using GraphPad Prism, version 9.

## Results

### Progenitor epithelial cells retain regenerative potential *in vitro* during physiological and accelerated aging

To evaluate the capacity of lung regeneration, epithelial cells were cultured in organoids, a model that allows its evaluation *in vitro* (Barkauskas et al., 2017). In brief, epithelial cells isolated from mice were mechanically and enzymatically dissociated and positively selected with the CD326 (EpCAM+) marker by Magnetic-Activated Cell Sorting (MACS). In this study, organoids were generated from 1,000 epithelial cells derived from young wild-type mice (12–16 weeks), physiologically aged wild-type mice (18–24 months), and Zmpste24-deficient mice, which exhibit accelerated aging (16 weeks) and with 10,000 pre-cultured fibroblasts from young wild-type mice (Figure 1a). This procedure yielded a cell population containing epithelial cells, including type II epithelial cells, as validated by the expression of surfactant protein C (Sftpc) through RT-qPCR and proSPC immunostaining (Figures 1b,c).

**FIGURE 1:**
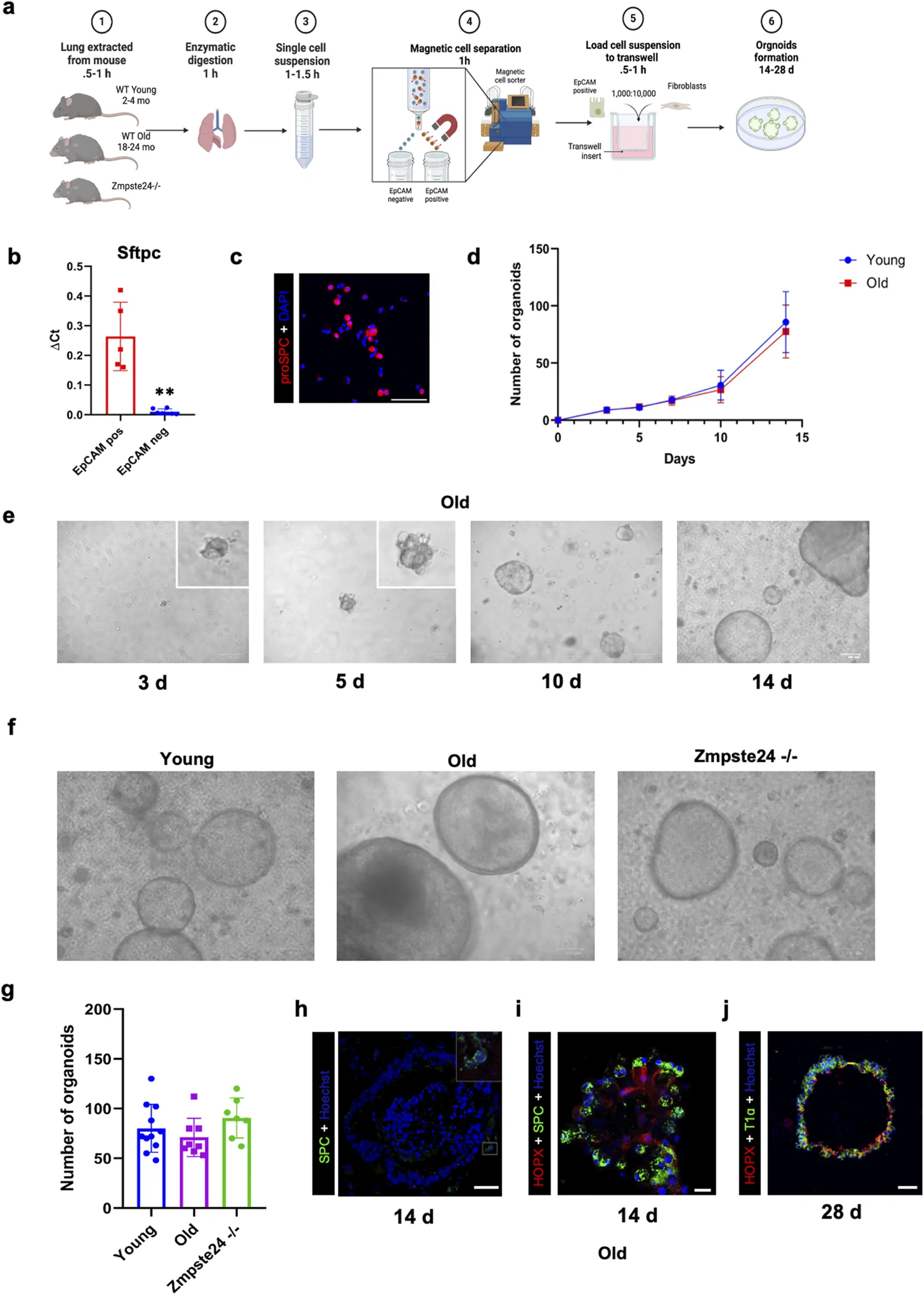
Lung progenitor epithelial cells retain organoid-forming potential during physiological and accelerated aging **(a)** Schematic representation of the experiment design. Created with BioRender.com. **(b)** RT-qPCR for the validation of the extraction of type 2 alveolar epithelial cells (AT2) obtained by magnetic beads (MACS) in wild-type mice by comparing the ΔCt of surfactant protein C (Sftpc) (EpCAM positive vs. negative fraction). Data are presented as mean ± SEM (n = 5) by triplicated (t-test **p < 0.01). **(c)** Representative immunofluorescence of proSPC in EpCAM+ fraction (Scale bar, 60 μm). **(d)** Quantification of organoid growth by age, where each data point represents one individual animal (young, *n* = 4; old WT, 24 months, *n* = 4). Statistical significance was determined using one-way ANOVA. **(e)** Brightfield micrographs showing organoids derived from 24-month-old WT mice at different days (d) of culture. Scale bar: 100 μm. **(f)** Representative brightfield micrographs of organoid cultures from wild type young, aged, and Zmpste24^−/−^ mice co-cultured with young WT fibroblast passage 3. (Scale bars, 100 μm). **(g)** Graph of organoids number where each data point represents one individual animal and corresponds to the mean of 2–4 technical replicate wells (n > 6) Statistical analysis was performed using one-way ANOVA followed by Tukey´s *post hoc* test. **(h)** Immunofluorescence staining for SPC in 14-day-old large basal organoids. Scale bar, 30 μm. **(i)** Staining for SPC and HOPX in 14-day-old organoids illustrating initial alveolar differentiation. Scale bar, 20 μm. **(j)** Staining for T1α and HOPX in 28-day-old organoids showing alveolar type I differentiation. Scale bar, 50 μm.

An organoid growth curve was performed with old and young wild-type cells, revealing a similar trend of increase (Figures 1d,e). Our results show that isolated epithelial cells from both aging models are capable of forming organoids in numbers and sizes comparable to those generated by young wild-type mice when co-cultured with fibroblasts from young wild-type mice (Figures 1f,g). After 2 weeks, the organoids displayed diverse multicellular structures with variations in size and morphology. As previously described, the type of organoid generated depends on the epithelial cell of origin. Larger rounded spheroids, likely corresponding to bronchiolar organoids, are characterized by smaller, more densely compacted cells, whereas alveolar organoids contain fewer cells and typically display an acinar organization (McQualter et al., 2010). Basal cells show significant pluripotency, being capable of regenerating both the airway epithelium and subsequently alveolar structures (Figure 1h) (Zepp and Morrisey, 2019). Furthermore, differentiation from AT2 into AT1 cells was confirmed by HOPX and T1α staining. These markers were observed in 14-day (SPC and HOPX) and 28-day (HOPX and T1α) organoids (Figure 1h).

### Fluorescence-activated cell sorting (FACS) impairs lung epithelial organoid-formation potential

In other studies, epithelial cells from physiologically old mice have been reported to form fewer organoids (Chanda et al., 2021; Rowbotham et al., 2023; Lehmann et al., 2020). A key difference between these studies and ours is that while previous work relied on FACS sorting to isolate epithelial cells, we used only magnetic bead-based isolation, which may influence regenerative outcomes. As shown in Figure 2a, incorporating an additional FACS EpCAM+ sorting step markedly prolongs processing time and exposes cells to shear stress. We therefore evaluated its impact on DNA damage and organoid formation. Under basal conditions, young cells show negligible γH2AX staining. Following flow cytometric sorting, γH2AX levels increase, suggesting that FACS induces DNA damage (Figures 2b,c). In contrast, aged cells exhibit higher basal γH2AX levels. Although γH2AX levels tend to increase further after sorting, no significant differences are observed relative to the basal levels in aged cells. As illustrated in Figures 2d-f, the incorporation of an additional FACS sorting step markedly reduced organoid growth under all conditions (Figures 2d-f). This effect was particularly pronounced in cells derived from aged mice, suggesting an increased susceptibility to the cellular stress associated with flow cytometric sorting, which may contribute to their reduced organoid-forming potential. Although FACS is commonly used to enhance cell purity, magnetic bead isolation alone yielded >85% purity in both young and aged samples (Supplementary Figure S2).

**FIGURE 2:**
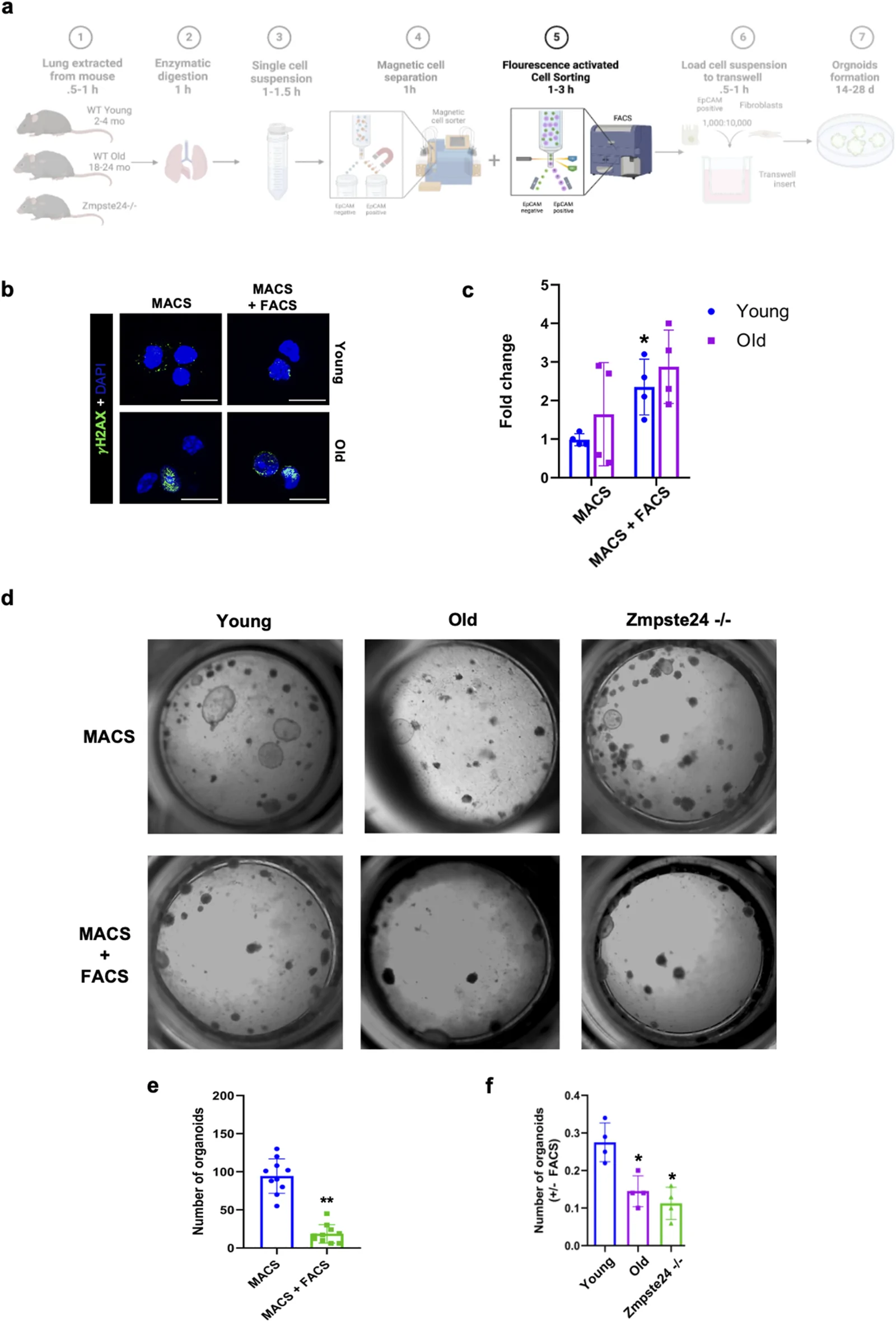
Magnetic-Activated Cell Sorting followed by Fluorescence-Activated Cell Sorting of epithelial cells reduces organoid growth. **(a)** Schematic representation of the experiment design. Created with BioRender.com. **(b)** Representative image of H2AX (green) immunofluorescence in EpCAM+ cells from old mice (24 months) and young, FACS-sorted versus unsorted (control) cells. Magnification: ×63. Scale bars 10 μm **(c)** Quantification of γH2AX immunofluorescence signal (fold change) in EpCAM+ cells from young and old mice, comparing FACS-EpCAM+ selection sorted and unsorted (control) groups. Statistical comparisons were made using ANOVA test (*p < 0.05). **(d)** Representative brightfield micrographs of organoid cultures (Scale bars, 100 μm). **(e)** Quantification of organoid number comparing FACS-sorted and unsorted (control) epithelial cells from different experimental groups to assess the effect of FACS independently of epithelial cell origin. Each data point represents one individual animal (n = 10 animals)t-test **p < 0.01). **(f)** Graph of organoid formation by age, data point represents one individual animal and corresponds to the mean of 2–4 technical replicate wells (young (n = 4), old WT 24 months (n = 4), and Zmpste24^−/−^ (n = 4) ratio comparing FACS-sorted versus unsorted (control) cells. Statistical comparisons were made using ANOVA test (*p < 0.05).

### Nuclear lamin loss during physiological and accelerated lung aging

To investigate the increased sensitivity of aged epithelial cells to flow cytometric sorting, we next examined age-related changes in nuclear lamins. Aging is known to alter the expression of nuclear lamins, contributing to stem cell dysfunction (Scaffidi and Misteli, 2008; Scaffidi and Misteli, 1979). However, these changes have not been characterized in the lung. In this study, we examined lamin A/C levels in lung tissues from young WT (n = 3), old WT (n = 3), and Zmpste24^−/−^ (n = 3) mice. Old mice displayed reduced lamin A/C levels throughout the lung. Quantification of fluorescence intensity, normalized to cell number, revealed a significant decrease in old WT and Zmpste24^*−/−*^ mice compared with young WT mice (Figures 3a,b). Immunoblotting of nuclear fractions for lamins A/C and B1 further corroborated these findings and showed that this decline occurs gradually with age (Figures 3c,d). Young mice exhibited higher levels of nuclear lamins than 14-month-old and 24-month-old mice, suggesting that age-related loss of nuclear lamins may compromise nuclear mechanics and increase cellular susceptibility to mechanical stress.

**FIGURE 3:**
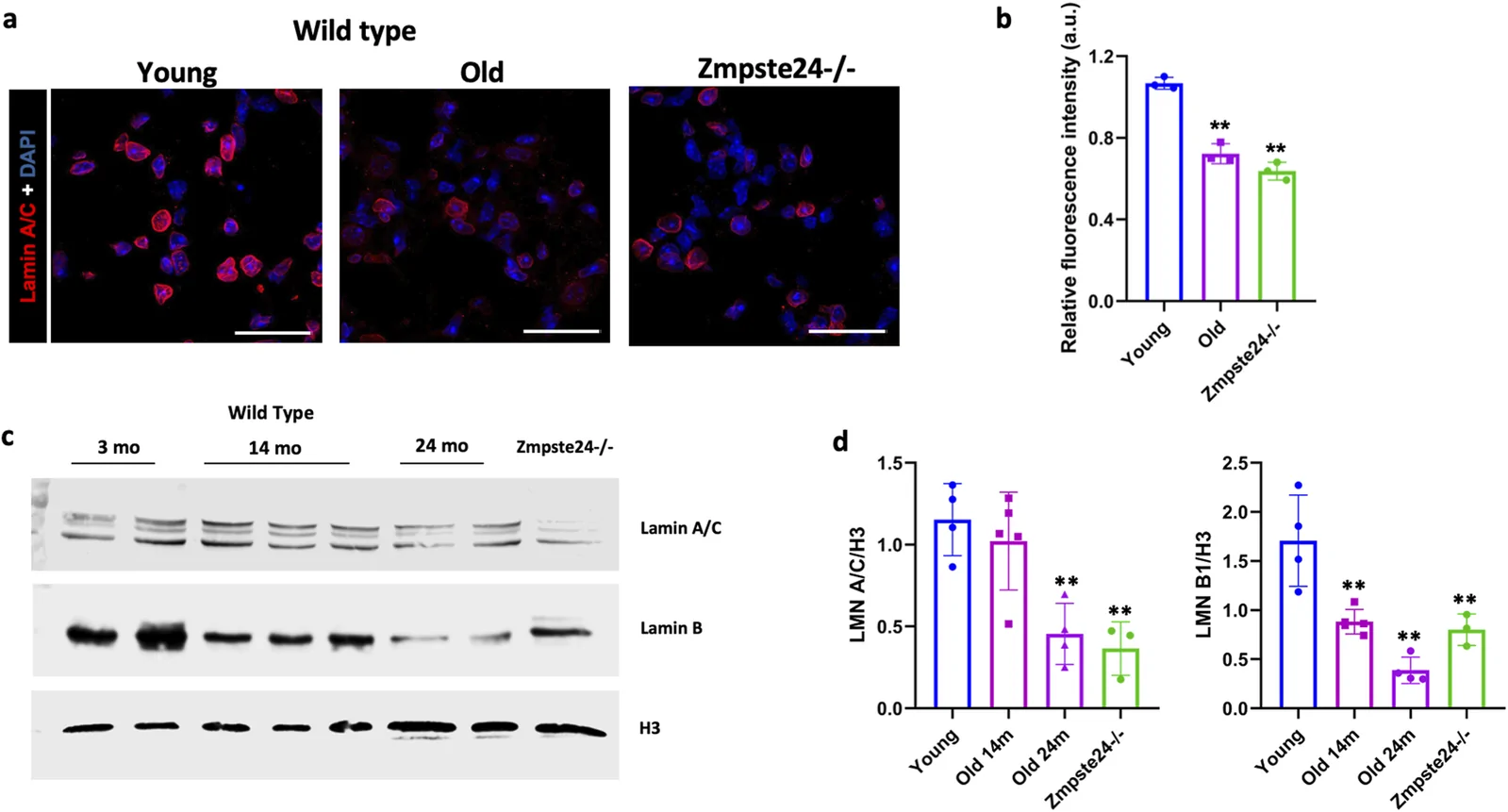
Nuclear lamins are decreased in lung tissue during physiological and accelerated aging. **(a)** Representative immunofluorescence images of lamin A/C in tissues from young, old, and Zmpste24^−/−^ mice (n = 3 mice per group) from three independent experiments, acquired at ×60 magnification with a 2.5 digital zoom. Scale bar: 20 μm. **(b)** Each data point in the graph represents an individual animal (n = 3) and corresponds to the average immunofluorescence analysis from at least four tissue fields per animal, acquired at ×40 magnification. The quantification of the mean fluorescence intensity of the nuclear lamina was normalized to the number of nuclei. Statistical analysis was performed using the ANOVA test, comparing each group to the control (**p < 0.01). **(c)** Representative immunoblot of Lamin A/C and B1 using H3 as a loading control in young (n = 3), old WT 14 months (n = 3), old WT 24 months (n = 2), and Zmpste24^−/−^ (n = 1) mice. Band intensity was quantified and normalized to H3. **(d)** Graph of the densitometric analysis performed on a total sample of young (n = 3), old WT 14 months (n = 3), old WT 24 months (n = 2), and Zmpste24^−/−^ (n = 1) mice, corresponding to two independent experiments. Statistical analysis was performed using one-way ANOVA.

### Aged epithelial cells maintain homeostasis through an autophagy-associated transcriptional response

Since autophagy plays a critical role in stem cell maintenance, we evaluated its related gene expression in EpCAM+ cells isolated from freshly digested mouse lungs. A two-dimensional hierarchical clustering analysis of 84 autophagy-related genes revealed that epithelial cells from aged wild-type and Zmpste24^−/−^ mice clustered together and separately from young wild-type cells (Figure 4a), indicating a shared aging-associated transcriptional profile.

**FIGURE 4:**
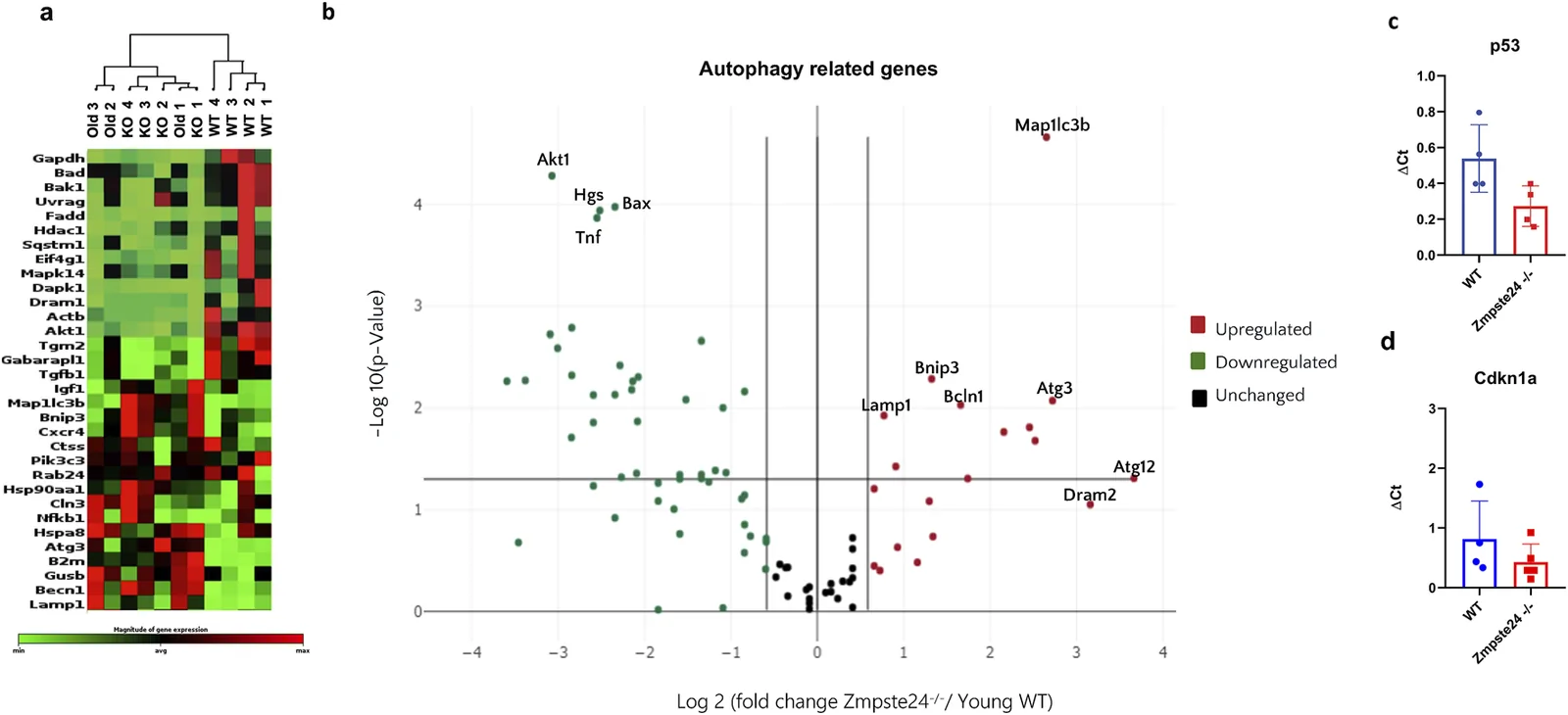
Epithelial progenitor cells show upregulation of autophagy-related gene expression but no changes in senescence markers during physiological and accelerated aging. **(a)** Two-dimensional hierarchical clustergram of 84 autophagy-related genes analyzed using the RT^2^ Profiler PCR Array Mouse Autophagy (PAMM-084Z, Qiagen). Samples correspond to freshly isolated EpCAM+ cells from Zmpste24^−/−^ (n = 4), old wild-type mice (n = 3), and young wild type mice (n = 4). Fold change ≥2; p < 0.05. **(b)** Volcano plot of autophagy genes of Zmpste24^−/−^ (n = 4) versus young WT mice (n = 4). Fold change 1.5. p-Value 0.05. **(c, d)** RT-qPCR for relative expression of CDKN1A and p53 in epithelial cells obtained by magnetic beads (MACS) in Zmpste24^−/−^ (n = 4) mice compared to wild-type mice (n = 4) where each data point represents the mean of three technical replicates from one individual animal.

Consistent with this observation, differential expression analysis showed increased expression of several autophagy-related genes, including LC3, Atg12, Atg3, and Lamp1, in epithelial cells from Zmpste24^−/−^ mice. In addition, Akt1, a negative regulator of autophagy, was significantly downregulated in Zmpste24^−/−^ cells (Figure 4b).

No significant differences were observed between EpCAM+ epithelial cells from Zmpste24^−/−^ and young wild-type mice (Figures 4c,d). Importantly, these analyses were performed on EpCAM+ cells isolated by magnetic bead separation without FACS sorting, minimizing the potential confounding effects of flow cytometry-induced stress. Together, these findings suggest that aged epithelial cells maintain cellular homeostasis through activation of autophagy-related pathways rather than by entering a senescent state.

### Senescent niche limits lung organoid growth

In the context of alveolar regeneration, substantial evidence supports the notion that facultative progenitor cells require a microenvironment or “niche” to promote their regenerative function (Yilmaz et al., 2012; Zepp et al., 2017). We found that isolated epithelial cells from Zmpste24^−/−^ and young WT mice show no difference in the number or organoid formation when co-cultured with mesenchymal cells derived from Zmpste24^−/−^ mice. In both cases, they form fewer, and smaller organoids (Figure 5).

**FIGURE 5:**
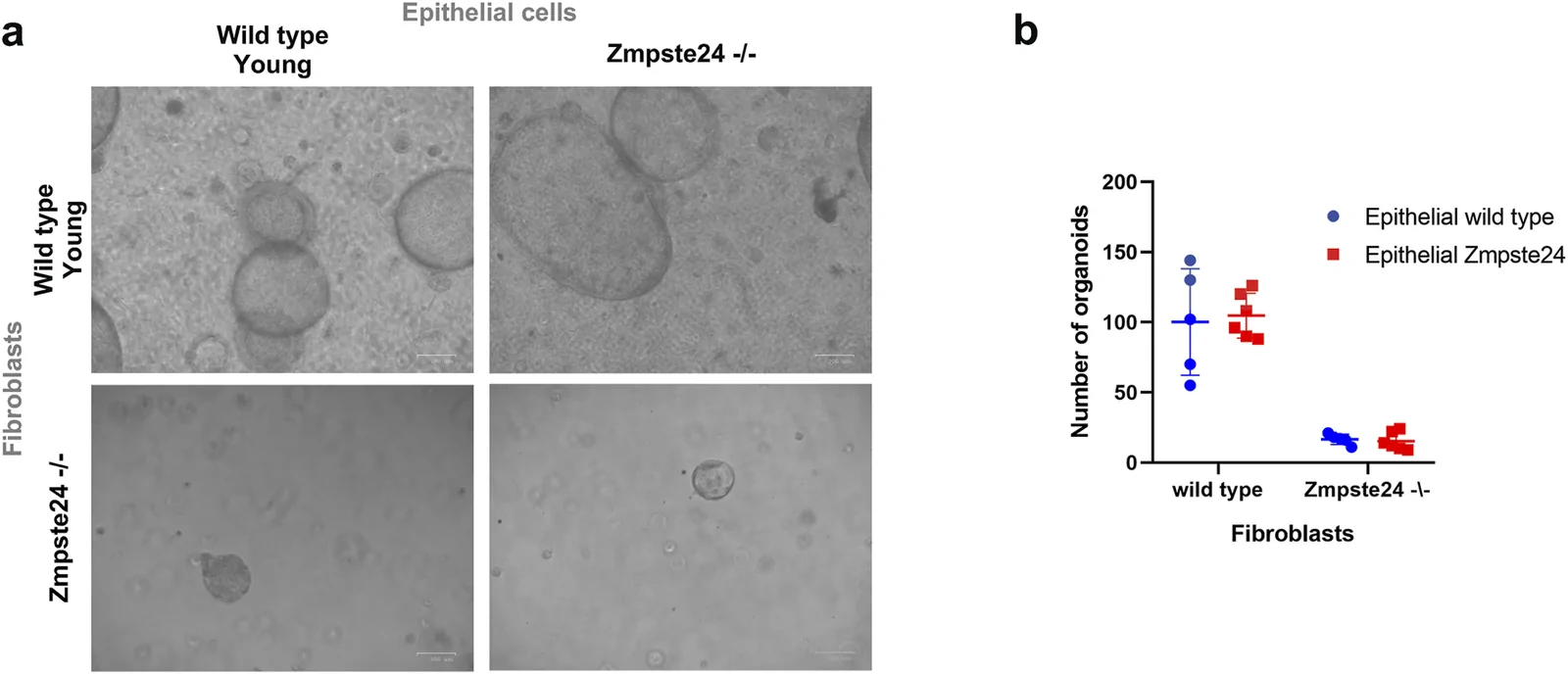
Organoids formation by epithelial cells co-cultured with Zmpste24^−/−^ mice fibroblasts. **(a)** Representative brightfield micrographs (Scale bars, 100 μm) of young WT and Zmpste24^−/−^ epithelial cells co-cultured with Zmpste24^−/−^ fibroblasts at passage 3. **(b)** Graph of organoids number. Data point represents one individual animal and corresponds to the mean of 2–4 technical replicate wells.

Therefore, we characterized the cellular state of the fibroblasts. Aged wild-type and Zmpste24^−/−^ (>16 weeks) fibroblasts showed a significant increase in nuclear deformations, such as bleb formation, compared to young controls (Figures 6a,b). Based on these similar nuclear alterations, and because Zmpste24^−/−^ fibroblasts offer a more homogeneous and tractable model for mechanistic studies, subsequent analyses of senescence, mTORC1 activation, and autophagy flux were performed in this model. Senescence was evaluated by SA-β-gal activity, which showed a significant increase of senescent cells in Zmpste24^−/−^ fibroblasts (Figures 6c,d). Moreover, we demonstrate that in Zmpste24^−/−^ fibroblasts the increase in senescence correlates with activation of the mTORC1 pathway, as evidenced by phosphorylation of pS6. S6 is a direct substrate of S6 kinase (S6K), which functions downstream of mTORC1, and its phosphorylation reflects activation of the mTORC1-S6K signaling axis (Figures 6e,f). By contrast, autophagy flux analyzed through LC3 and p62 after chloroquine (CQ) treatment, was decreased in these cells (Figures 6g,h), suggesting that fibroblasts reach senescence with a decrease in autophagy compared to young WT. In Zmpste24^−/−^ mice, fibroblast senescence is irreversible, even when treated with the pharmacological inhibitor of mTOR Rapamycin for 24 h (Supplementary Figure S3). To confirm that Zmpste24^−/−^ fibroblasts were senescent rather than quiescent, the proliferation marker Ki67 was evaluated, revealing a lower expression of this marker in Zmpste24^−/−^ fibroblasts compared to WT cells (Supplementary Figure S3).

**FIGURE 6:**
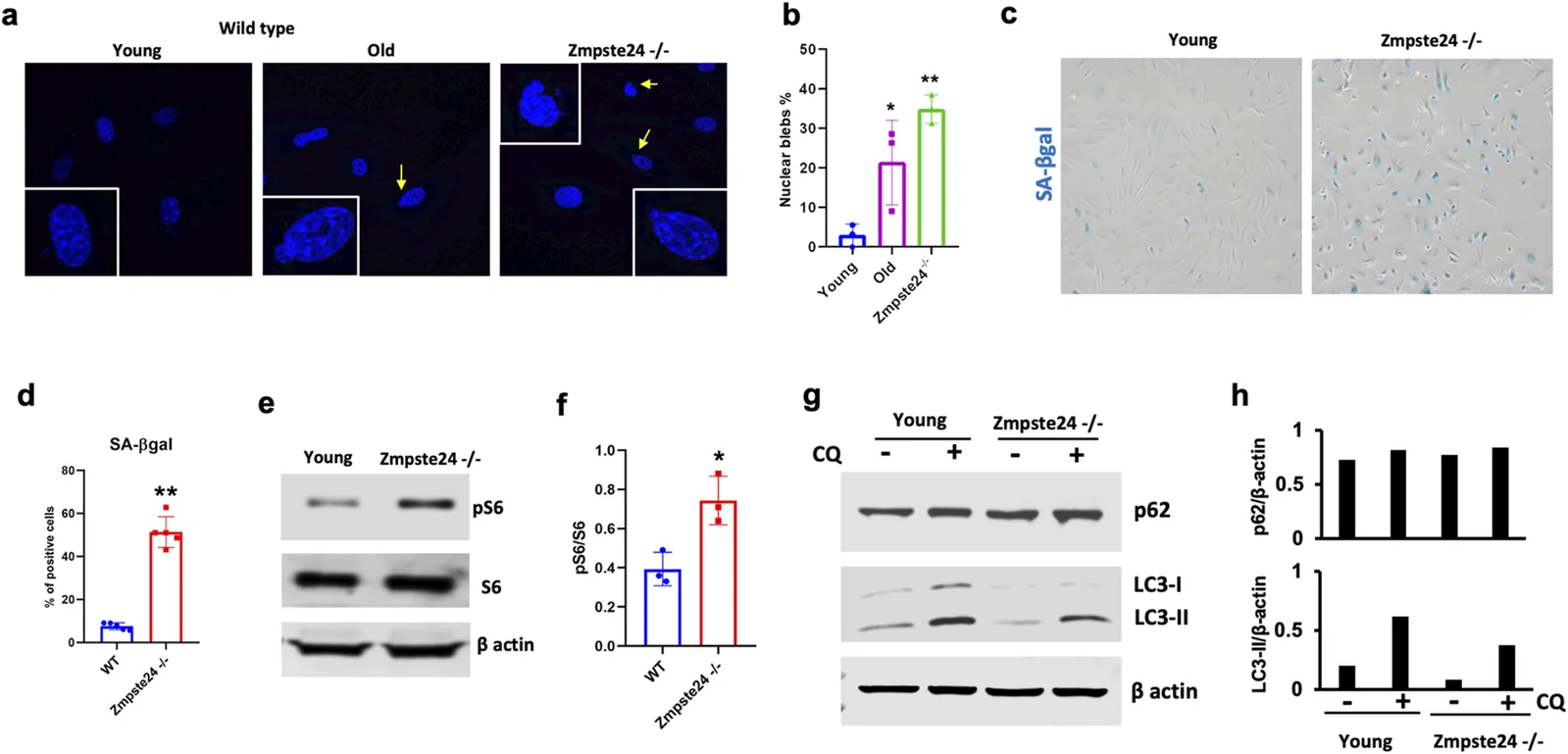
Fibroblasts from aged mice exhibit nuclear defects and, in the Zmpste24^−/−^ model, acquire a senescent phenotype characterized by mTORC1 activation and impaired autophagy. **(a)** Representative image of fibroblast nuclei from young and old WT and Zmpste24^−/−^ mice, of three animals per condition. Arrowheads indicate sites of nuclear rupture. **(b)** Quantification of nuclei exhibiting blebs, presented as the mean percentage of nuclei analyzed from three independent animals per condition. Statistical analysis was performed using one-way ANOVA. **(c)** Representative image of fibroblasts from Zmpste24^−/−^ mice (older than 16 weeks) show significant increase of Senescence Associated -β galactosidase (SA-β-gal) in fibroblasts from Zmpste24^−/−^ mice and young WT at passage 3. **(d)** Graph of percentage SA-β-gal positive cells, each data point represents one individual animal, calculated from at least three fields per animal (n = 5 animals per condition, t-test **p < 0.01). **(e)** Representative immunoblot of activity of mTORC1 by phosphoS6 and S6 in mesenchymal cells from aged mice Zmpste24^−/−^ versus WT (β-actin as a loading control). **(f)** Graph of pS6/total S6 ratio (n = 3, t-test *p < 0.05). **(g)** Representative immunoblot for autophagy flux: LC3 and p62 with/without chloroquine 10uM (CQ) for 24 h in fibroblasts from Zmpste24^−/−^ mice and WT with β-actin as a loading control (one representative of two independent experiments is shown n = 3). **(h)** Densitometric analysis of p62 and LC3-II levels normalized to β-actin.

We induced mTORC1 activation in young wild-type fibroblasts through a combination of starvation and nutrient stimulation in the presence of chloroquine. This dual treatment promotes lysosomal biogenesis and changes in lysosomal acidity resulting in mTORC1 activation (Zoncu et al., 1979). Figure 7a corroborates that this treatment activates mTORC1 signaling, as indicated by elevated levels of phosphorylated S6. Organoid culture with fibroblasts with this double treatment as well as fibroblasts from Zmpste24^−/−^ forms fewer, and smaller organoids compared to control fibroblasts (Figures 7b,c). Thus, when autophagy was inhibited and mTORC1 activity increased, the organoids grew similarly as those cultured with KO fibroblasts. Furthermore, we observed senescence in these organoids. When co-cultured with wild-type fibroblasts subjected to the double-hit treatment or with Zmpste24^−/−^ fibroblasts, the organoids also displayed senescent cells (Figure 7c).

**FIGURE 7:**
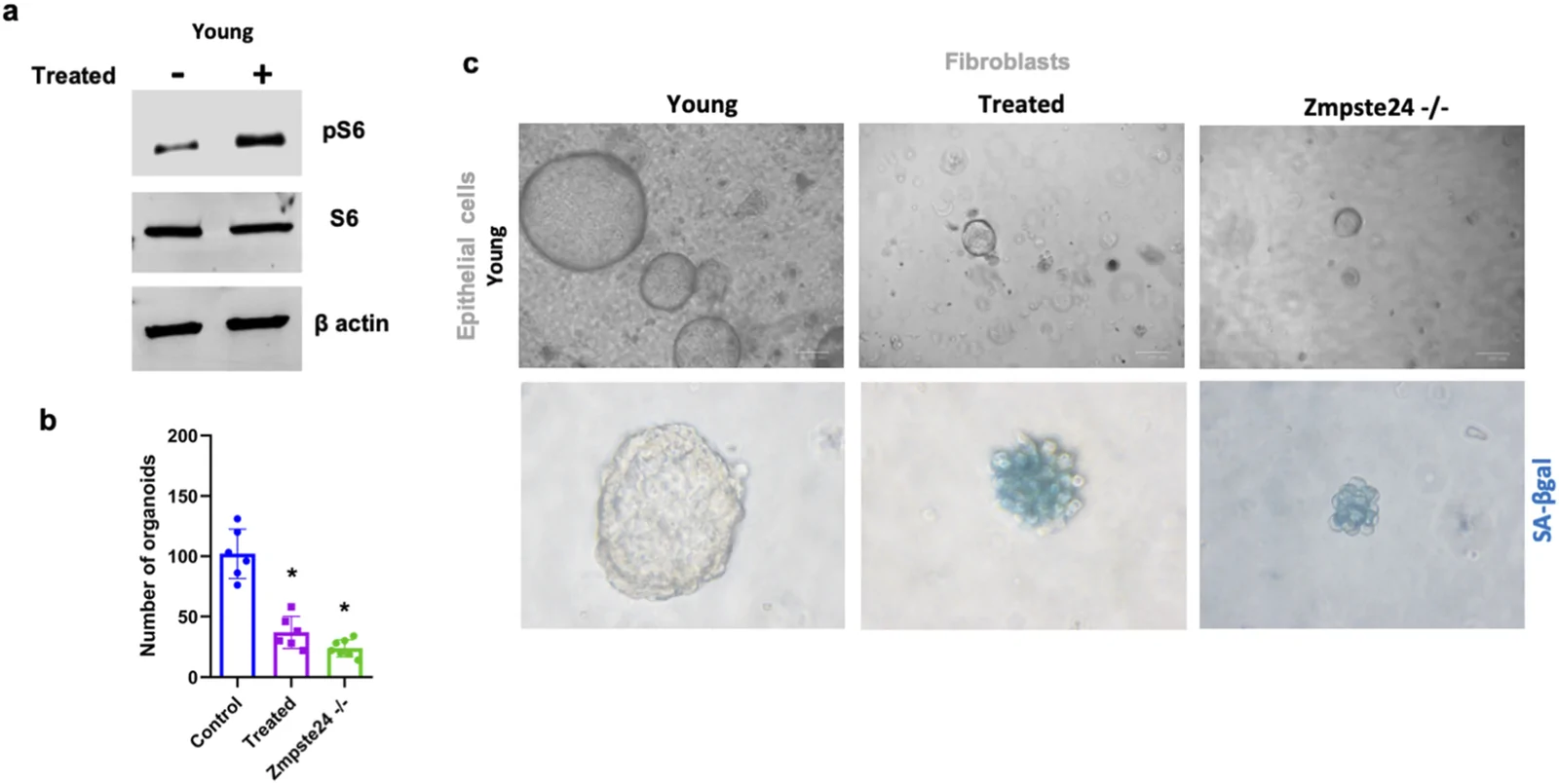
Fibroblasts from wild type mice with increased mTORC1 activity and from Zmpste24^−/−^ mice prevent organoid formation by inducing senescence. **(a)** Representative Western blot shows that fibroblasts from wild-type mice exhibited mTORC1 activity by increased levels of S6 phosphorylation, these fibroblasts were treated with nutrient deprivation (HBSS, 3 h) and chloroquine (10 μM) for 24 h **(b)** Number of organoids generated from young wild-type epithelial cells co-cultured with young fibroblasts control and treated with: HBSS for 3 h followed by CQ (10 μM) for 24 h, or with Zmpste24^−/−^ fibroblasts. Statistical comparisons were made using ANOVA test (n = 6 animals per condition, *p < 0.05) from two independent experiments. **(c)** Representative brightfield micrographs of organoids and senescence associated -β galactosidase (SA-β-gal) stain.

## Discussion

Aging increases the morbidity and mortality associated with respiratory diseases such as chronic obstructive pulmonary disease and idiopathic pulmonary fibrosis. Contributing factors include stem cell exhaustion and altered intercellular communication, which are linked to limited regenerative capacity (López-Otín et al., 2013; López-Otín et al., 2023). Thus, comprehending the alterations in stem cell-niche interactions is critical for understanding regeneration during aging. To investigate this, we used two models: aged wild-type mice and an accelerated-aging mouse model.

Regeneration was assessed using three-dimensional lung organoid cultures, which mimic the cell interactions of the lung and provide a platform to assess regenerative capacity (McQualter et al., 2010).

Our results demonstrate that lung epithelial cells from both aged and Zmpste24^−/−^ mice maintain regenerative capacity. To investigate the mechanisms underlying this preserved function, we further characterized the cellular state of these epithelial cells. We found that epithelial cells are not senescent and maintain their lung progenitor phenotype, displaying an autophagy-associated response, as assessed by gene expression analysis. Although we were unable to directly assess autophagic flux in epithelial cells, due to the potential loss of their epithelial phenotype during the required *in vitro* manipulation, this remains a limitation of the present study. Therefore, we cannot conclude that autophagic flux is increased, but rather that these cells exhibit an autophagy-related transcriptional response. We speculate that these cells may have developed an adaptive mechanism through which autophagy contributes to the maintenance of stem cell homeostasis. Paradoxically, in Zmpste24-deficient mice, increased autophagy has been described as a systemic feature; however, this elevation does not delay aging in other organs as it appears to do in the lung (Marino et al., 2014). Consistent with this, our cluster analysis revealed that genes related to autophagy exhibit a similar expression pattern in Zmpste24^−/−^ and aged WT mice, suggesting that this response may be a common feature of physiological aging. This convergence could be explained, at least in part, by the intrinsically low cell turnover of the lung parenchyma (Rando, 2006). Moreover, Zmpste24^−/−^ mice have been reported to exhibit a protective response to bleomycin-induced fibrosis (Calyeca et al., 2018). Taken together, these findings support the notion that aging may trigger a hormetic response in the lung, characterized by resistance to senescence and activation of autophagy.

In this study, we also demonstrated that the expression of nuclear lamins A/C and B1 progressively decreases in the lung during both physiological aging and in the accelerated aging model Zmpste24^−/−^. This finding is relevant, as nuclear lamins are essential for maintaining nuclear structural integrity and resisting deformation; consequently, their reduction increases vulnerability to mechanical stress. In accelerated aging models such as Hutchinson–Gilford progeria syndrome (HGPS), alterations in lamin A have been shown to induce defects in chromatin organization, loss of perinuclear heterochromatin, and increased DNA damage (Scaffidi and Misteli, 1979). Furthermore, reduced lamin A/C expression has been associated with nuclear reorganization characterized by the loss of lamin-associated heterochromatin domains (LADs), alterations in epigenetic marks, and disruption of three-dimensional genome architecture, with direct consequences for gene expression and tissue regeneration (Jin et al., 2024; Wang et al., 2022). Similarly, our results suggest that the progressive loss of nuclear lamins in the aged lung may compromise regenerative capacity *in vitro* (Scaffidi and Misteli, 2008; Meshorer and Misteli, 2006).

Although this link between lamin loss and progenitor cell status has not been previously studied in the lung, our findings provide initial evidence supporting a potential association. We observed that the inclusion of a FACS step not only prolonged processing time but also was also associated with increased DNA damage (γH2AX) and significantly reduced organoid formation, with this effect being more pronounced in aged cells, in which lamin loss may affect nuclear recovery capacity. These results are consistent with the phenomenon described as sorter-induced cell stress (SICS), which encompasses shear forces, pressure fluctuations, abrupt accelerations, and electrical charges during sorting, all of which exceed the mechanical tolerance of cells (Shah et al., 2021; Maremonti et al., 2022; Brindley et al., 2011; Pfister et al., 2020; Ryan et al., 2021; Choudhury et al., 2024). When comparing cell isolation methods, we found that MACS yielded highly pure cells that displayed better preserving organoid-forming capacity, whereas FACS, despite offering higher resolution, was associated with reduced organoid formation potentially due in part to procedure-associated mechanical stress. Although the efficacy of MACS depends on proper optimization of antibody and bead labeling, it generally represents a faster and less damaging alternative for isolating cells (Sutermaster and Darling, 2019) Taken together, our findings support the hypothesis that the reduction of nuclear lamins during lung aging increases susceptibility to mechanical stress, and that technical factors such as the use of FACS may exacerbate this vulnerability. These results underscore the importance of standardizing isolation methodologies in lung organoid studies and highlight the need to further investigate lamin–chromatin dynamics and their interaction with mechanical stimuli.

Age-related decline in the lung cannot be explained solely by stem cell exhaustion but also involves pro-aging factors originating from the niche (Chakkalakal et al., 2012) While epithelial cells in the lung encompass multiple progenitor populations, in the alveolar context our focus is on alveolar epithelial type 2 cells and their specific niche formed by adjacent fibroblasts. These facultative progenitors play a central role in maintaining alveolar homeostasis and regeneration through close epithelial-mesenchymal interactions (Liberti and Morrisey, 2021). Our findings show that fibroblasts derived from physiologically aged and Zmpste24^−/−^ mice exhibit nuclear defects and a senescent phenotype characterized by impaired autophagy and sustained activation of mTORC1 signaling. When co-cultured with epithelial cells, organoid formation was markedly impaired. These findings are consistent with those reported by Chanda *et al.*, who found that fibroblasts from 24-month-old mice limit epithelial organoid growth (Chanda et al., 2021). In the same line, in human lung fibroblasts, we previously reported a persistent activation of mTORC1, and a decreased autophagy associated with aging (Romero et al., 2016). While mTOR activation has been implicated in promoting senescence, its causal role in aging within the alveolar niche remains to be established (Carroll et al., 2017; Narita et al., 2011). Additionally, short-term pharmacological inhibition of mTOR with rapamycin for 24 h was insufficient to reverse fibroblast senescence phenotype under the conditions tested, indicating that further genetic or pharmacological approaches will be required to clarify the contribution of mTOR signaling to this phenotype.

A fundamental question in the biology of aging is why aging progresses at different rates within the same organism, affecting organs, tissues, and cells unequally. In the adult lung, for example, it remains unclear how the number and functional capacity of adult stem cells change with age (Schneider et al., 2021) In one of the models we used, aging is caused by a defect in the nuclear lamina, like progeria syndrome, with the phenotype developing gradually over time (Pendás et al., 2002). In our study, we have corroborated that despite having Zmpste24 deletion (at 16 weeks of age), freshly isolated Zmpste24^−/−^ fibroblasts retain a proliferative capacity comparable to that of wild type. However, nuclear defects become evident by passage 3, coinciding with the onset of cellular senescence. These results confirm that aging, even linked to a genetic defect, depends on the development of specific mechanisms. Therefore, it is necessary to consider the influence of external factors such as matrix stiffness, particularly in aging, where mechanical stress may exacerbate nuclear alterations (Toscano-Marquez et al., 2023). In this regard, it has been proposed that during the process defined as *fibroaging*, cells undergo changes in the cellular microenvironment, receiving mechanical stimuli such as extracellular matrix stiffness, which leads to a reorganization of the cytoskeleton and induces changes in nuclear architecture, resulting in epigenetic reprogramming (Selman and Pardo, 2021). ECM changes have been considered as a new integrative hallmark of aging (López-Otín et al., 2023). In WT mice aging is associated with the upregulation of several ECM proteins. Interestingly, although fibrillar collagen genes are not upregulated in Zmpste24^−/−^ mice, lung collagen content shows a similar increase with aging, suggesting that the accumulation of this protein characteristic of aging occurs despite lamin A defects (Calyeca et al., 2018). However, a potential limitation of our study is that Zmpste24^−/−^ although it recapitulates key features of premature aging, it does not fully represent the complex and multifactorial nature of physiological aging. Additionally, it is important to acknowledge that, like any *in vitro* model, organoids have inherent limitations, such as the absence of vascularization, immune components, and mechanical signals associated with respiration, that restrict the direct extrapolation of findings to *in vivo* lung regeneration. Therefore, *in vivo* studies will be necessary to validate these observations (Mao et al., 2026).

In summary, these findings support a close interplay between progenitor cells and their niche, in which microenvironmental signals coordinate an autophagy-driven stress response to promote regeneration. Understanding the mechanisms by which these cells lose function with aging will provide deeper insight into their role in the pathophysiology of various respiratory diseases.

## Data Availability

The original contributions presented in the study are included in the article/Supplementary Material, further inquiries can be directed to the corresponding author.
