# Supplementary material for: Aging remodels progenitor and niche interactions in lung regeneration
Source: Front Aging. 2026 Oct 28;7:1986986. doi: 10.3389/fragi.2026.1986986 (PMC13751513; doi:10.3389/fragi.2026.1986986)
Supplement: Supplementary file 1 [file DataSheet1.pdf]

a

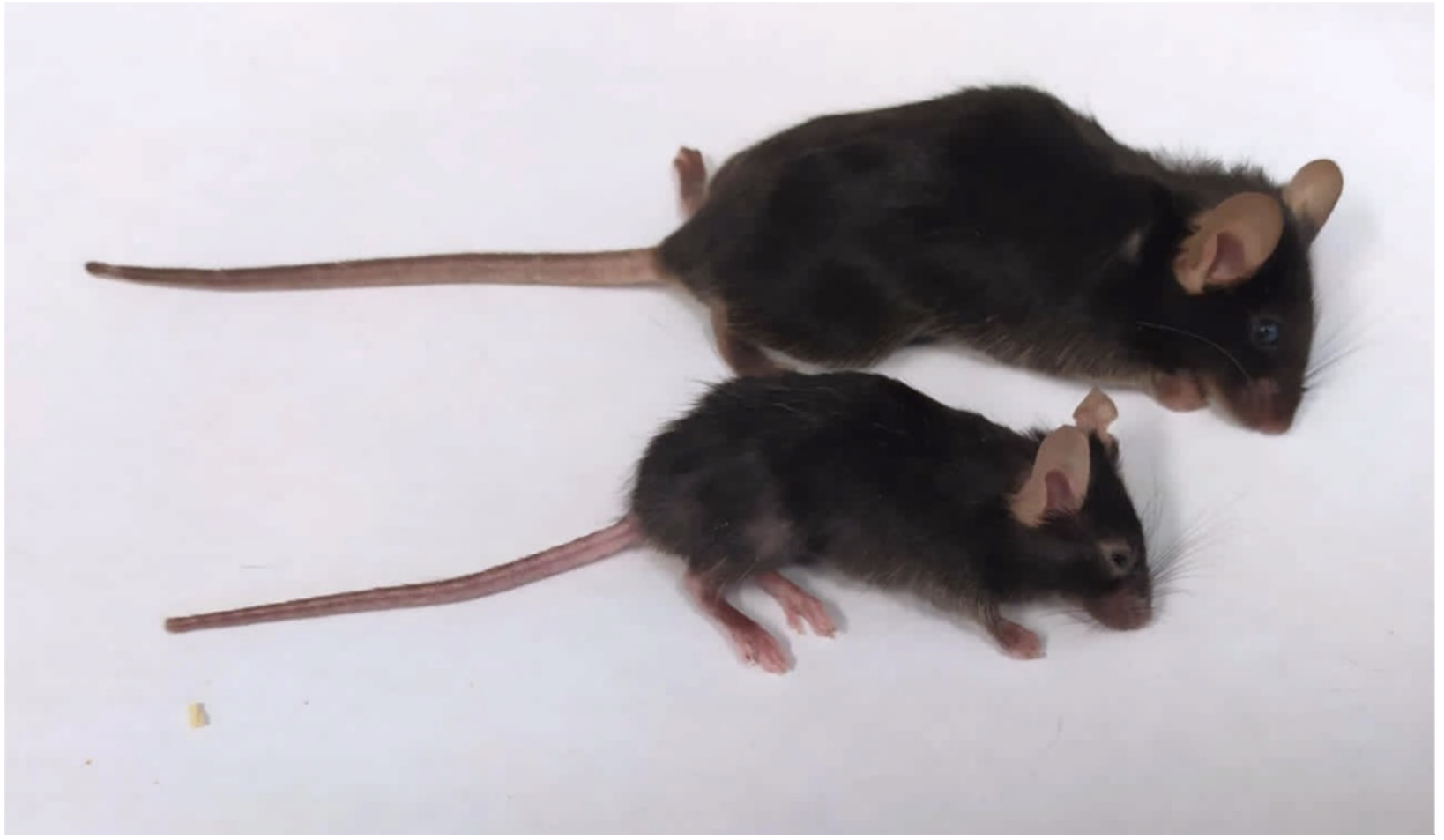

b

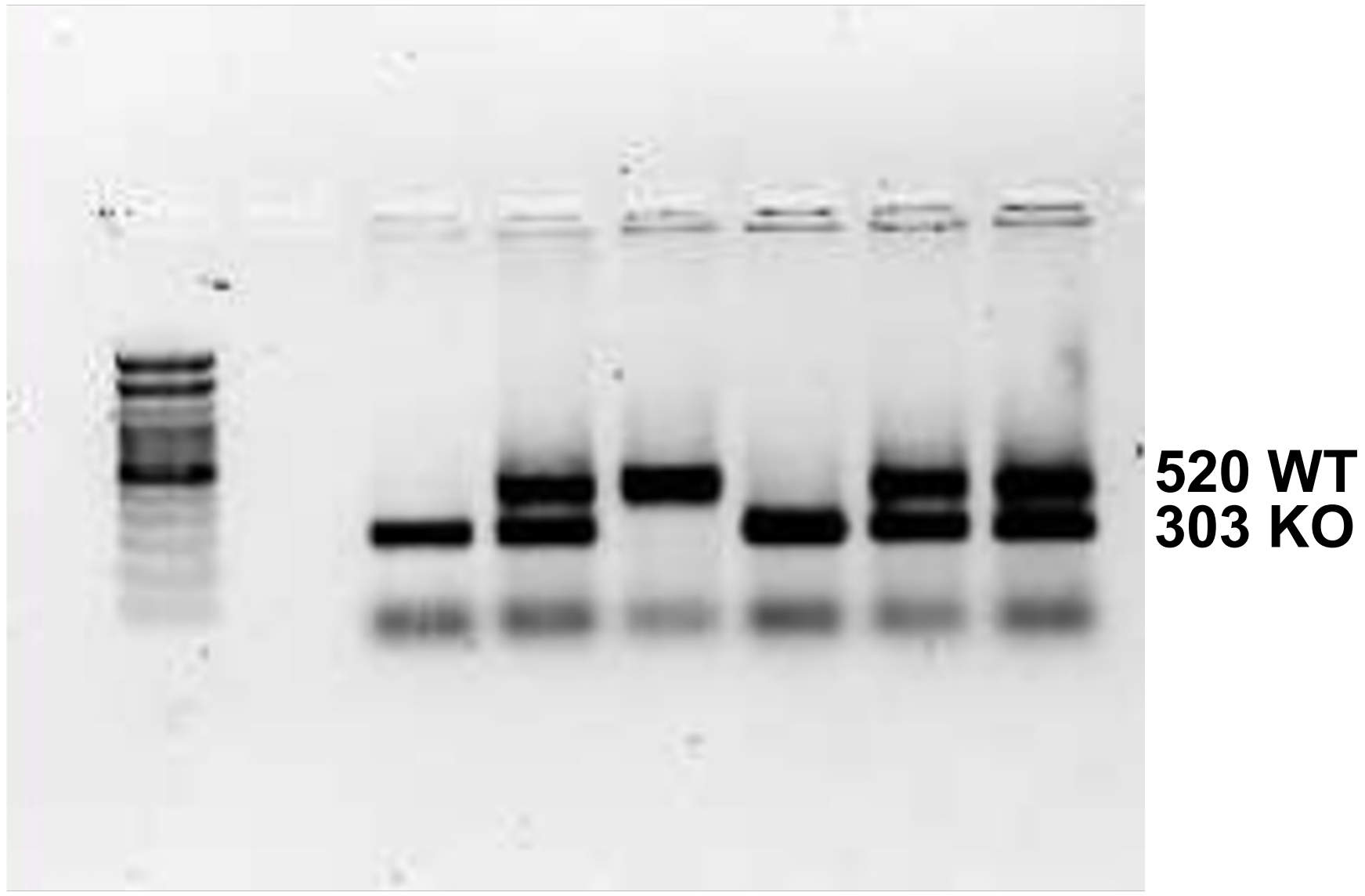

Supplementary figure. 1

Wild type young

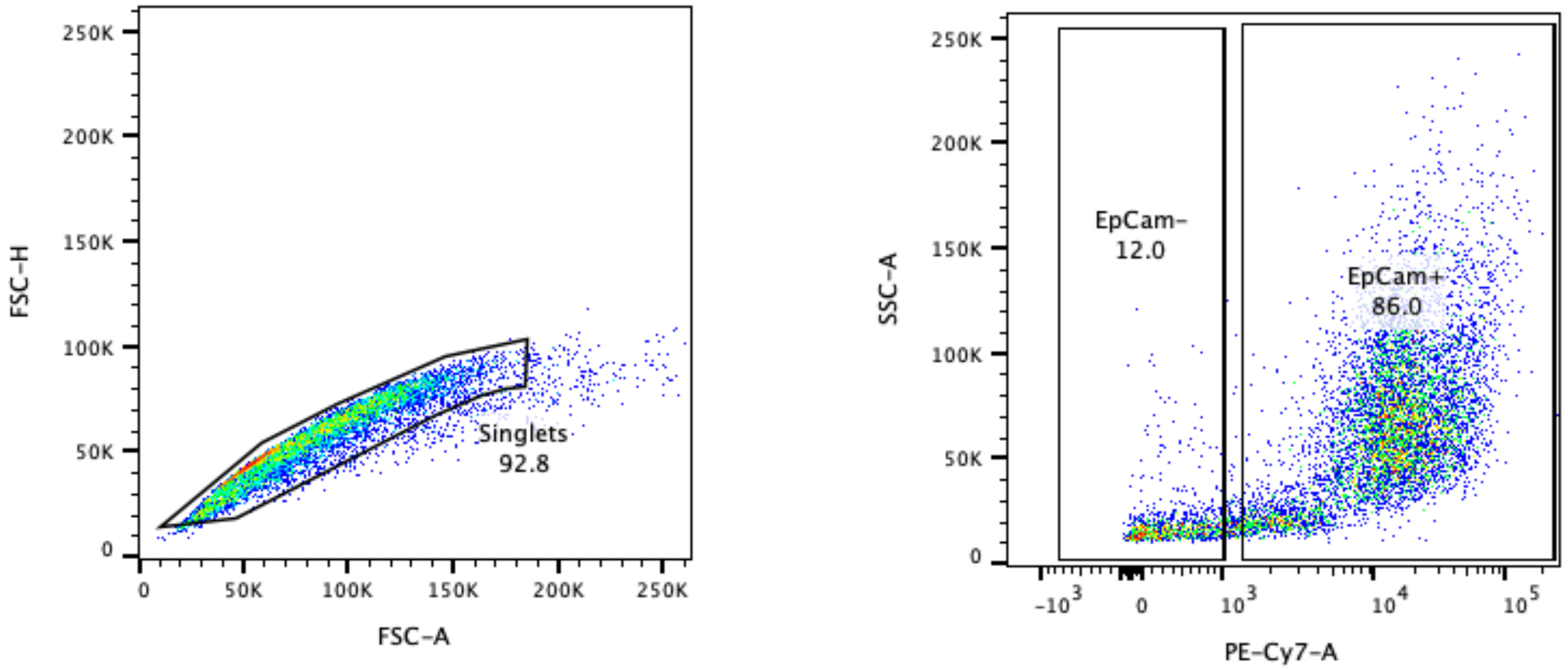

Wild type old

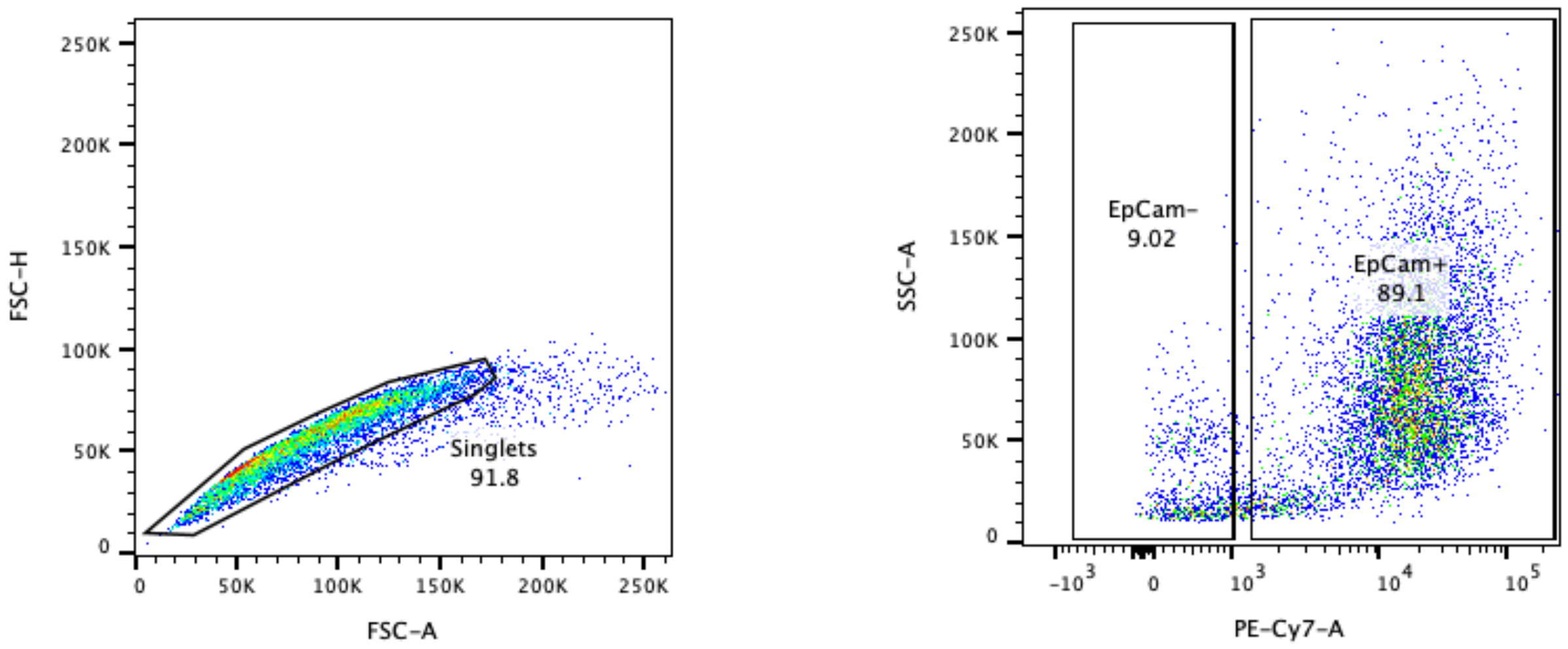

Supplementary figure. 2

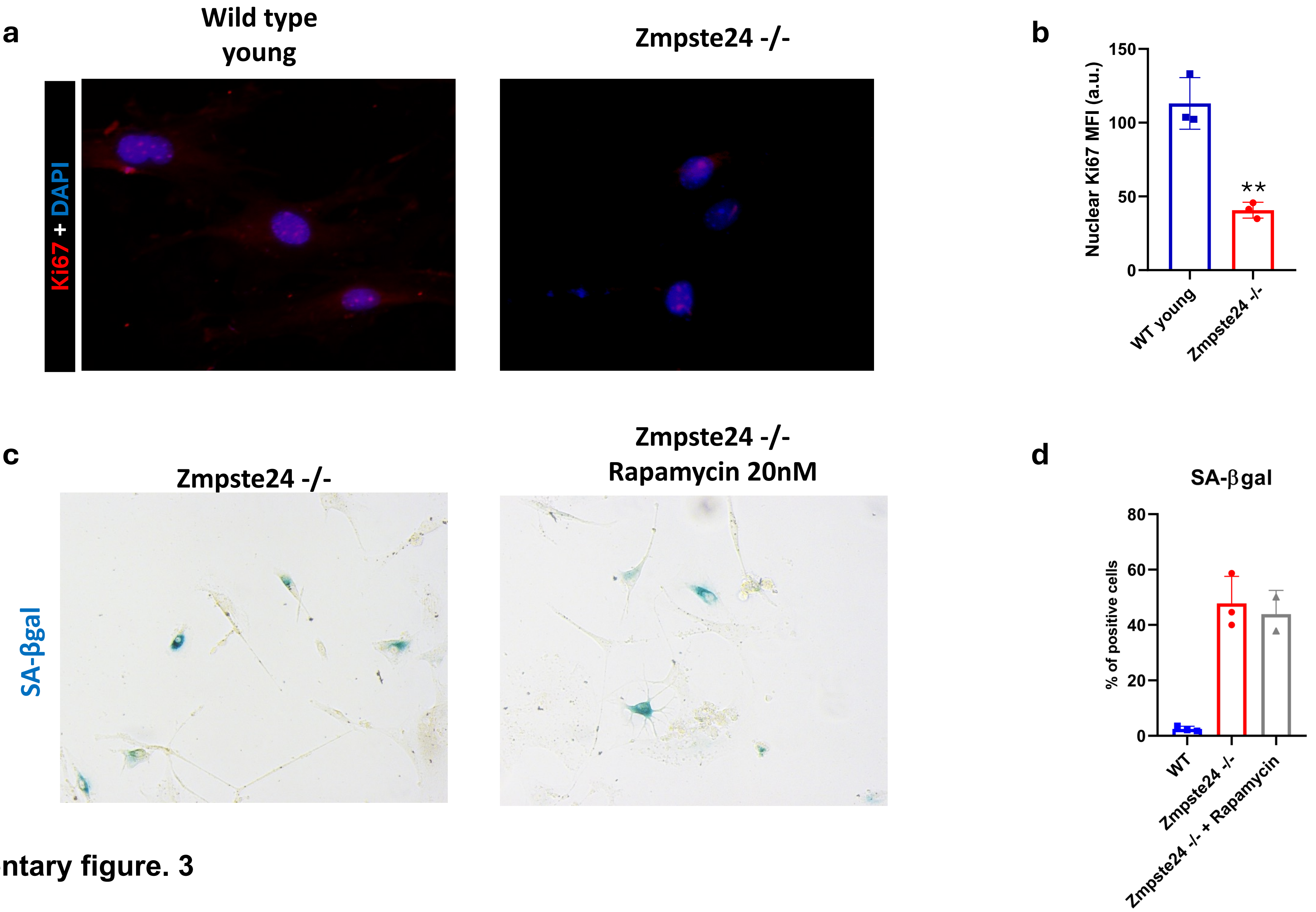

Supplementary figure. 3
